# Anæsthetic Revelations

**Published:** 1886-08

**Authors:** 


### *ANÆSTHETIC REVELATIONS.*

"Anæsthetic Revelations and the Gist of Prophecy" is the title of a work by an American author who asserts that "there is an invariable and reliable condition ensuing about the instant of recall from anæstine stupor to sensible observation in which the genius of being is revealed."

The author having presented a copy of his work to Mr. Tennyson, the English Poet Laureate, with whose works our readers have an intimate acquaintance, received from him a formal acknowledgement in which the following language occurs:

"I have never had any revelations through anæsthetism, but a kind of 'waking trance,' (this for lack of a better word.) I have frequently had quite up from boyhood, when I have been alone. This has often come upon me through repeating my own name to myself, silently, till all at once, as it were out of the intensity of consciousness of individuality, the individuality itself seemed to dissolve and fade away into boundless being—and this not a confused state, but the clearest of the clearest, the surest of the surest, utterly beyond words, whose death was an almost laughable impossibility—the loss of personality (if so it were) seeming no extinction, but only true life."

"I am ashamed of my feeble description. Have I not said the state is beyond words? But in a moment, when I come back into my normal condition of sanity, I am ready to fight for 'Meine Liebe Ich' and hold that it will last for æons of æons."

The letter of Tennyson is dated May 7th, 1874.

---

**A Correspondent** writes that with HALL'S JOURNAL in hand, he "can almost fancy himself reading the *Banner of Light*." If this is meant as a compliment,well and good. The *Banner* is one of the most valued of our exchange, from whose well stored columns we never fail to glean many beautiful thoughts, that tend to the world's moral and intellectual advancement, all the more valued for being so far removed from the common place. The day has gone by when a monoply of truth whether scientific, secular or theologic is to be conceded as the exclusive inheritance of any class or sect. Let us by all means have *agitation* rather than *stagnation* upon all the great questions of the day which relate to human affairs. We shall never cease to have a hearty good word for the intellectual pioneers who enter new fields of thoughts only to expose error and make the way smooth for the acquirement of knowledge, which is the growth of the spirit.

---

**The first number** of the *Practical Physician* published at 83 Elm St., New York City, has been laid upon our table. We gather ftom its introductory that its purpose is "to disseminate among the people a knowledge of the philosophy of cure employed by the new school of practical physicians" who claim the gift of healing to be an actual human endowment irrespective of the use of drugs and materia medica as taught in established medical institutions. We wish the *Practical Physician* every success in this new interprise.

---

**Family** physician meets patron on the street: "Ah, good morning, Mr. Simpson, how is your good wife to-day?" "She's ever so much better, sir—a little restless the fore part of the night, but very quiet afterwards." "Yes, yes, that's encouraging; my new preparation is sweeping all before it; don't you think so Mr. Simpson?" "I know it, sir. By the by, Dr. Dosem, the funeral is appointed for to-morrow afternoon; may I expect you to be present?"

